# Circulating Bone morphogenetic protein 9 (BMP9) as a new biomarker for noninvasive stratification of nonalcoholic fatty liver disease and metabolic syndrome

**DOI:** 10.1007/s10238-024-01316-0

**Published:** 2024-03-16

**Authors:** Yuchen Yang, Meihong Gu, Wei Wang, Shan Li, Jinlai Lu, Qinjuan Sun, Miao Hu, Lan Zhong

**Affiliations:** 1 Department of Gastroenterology, Shanghai East Hospital, Tongji University School of Medicine, No 150, Jimo Road, Pudong New Area, Shanghai, 200120 China; 2 Department of Gastroenterology, The Second Hospital of PingHu, Jiaxin, 314201 China

**Keywords:** Nonalcoholic fatty liver disease, Bone morphogenetic protein 9, Metabolic syndrome, Nonalcoholic steatohepatitis, Risk factor

## Abstract

Nonalcoholic fatty liver disease (NAFLD) is closely related to metabolic syndrome (MetS). Bone morphogenetic protein 9 (BMP9) is an essential factor in glucose, lipid and energy metabolism. This study aims to investigate whether BMP9 can serve as a serological marker for the severity of NAFLD or MetS. Blood samples, clinical data and FibroTouch test were collected from consecutively recruited 263 individuals in Shanghai East hospital. All the participants were divided into three groups: the healthy controls, nonalcoholic fatty liver (NAFL) group and nonalcoholic steatohepatitis (NASH) at-risk group according to the results of FibroTouch test and liver function. Serum BMP9 levels were measured by enzyme-linked immunosorbent assay. Serum BMP9 levels were positively correlated with transaminase, triglyceride, fasting plasma glucose, glycated hemoglobin (HbA1c) and uric acid while it showed a downward trend as the increasing number of MetS components. Furthermore, it differentiated NASH at-risk (58.13 ± 2.82 ng/L) from the other groups: healthy control (70.32 ± 3.70 ng/L) and NAFL (64.34 ± 4.76 ng/L) (*p* < 0.0001). Controlled attenuation parameter of liver fat and liver stiffness measurement were negatively correlated with BMP9 levels, while high-density lipoprotein levels were positively correlated. The risk of developing NAFLD increased along with elevated serum BMP9 and BMI, and a significantly higher risk was observed in men compared to women. BMP9 should be considered a protective factor for the onset and development of NAFLD, as well as a promising biomarker for the severity of the NAFLD and MetS.

## Introduction

Nonalcoholic fatty liver disease (NAFLD) is a spectrum of liver disease, including simple hepatic steatosis, nonalcoholic steatohepatitis (NASH), liver cirrhosis and even hepatocellular carcinoma (HCC). NAFLD has become the most common chronic liver disease in the world, with a global comprehensive prevalence of 25% and an increasing trend year by year [1]. It is closely associated with metabolic syndrome (MetS) including obesity, hypertension, hyperglycemia and dyslipidemia [2]. NAFLD may be both the liver manifestation and the cause of MetS [3], and there is an interplay between these two diseases. Recently, NAFLD with metabolic disorders has been redefined as metabolic-associated fatty liver disease (MAFLD) [4]. NASH is an inflammatory progressive form of NAFLD and can lead to cirrhosis and HCC without timely intervention. Recognition and diagnosis of different stages, particularly NASH, are very important for the management of NAFLD. Although liver biopsy is the gold standard for the diagnosis and the monitoring of NAFLD, the limitation is not only that it is an invasive procedure due to trauma and complications such as bleeding and bile fistula risks, but also its expensive nature of liver biopsy-based diagnosis, sampling and inter pathologist variations [5]. Therefore, there is an unmet need for seeking brand-new noninvasive techniques or biomarkers that can be used not only to detect NASH among patients with metabolic risk factors or MetS, but also to monitor the effect of treatment.

Bone morphogenetic protein 9 (BMP9), a member of the transforming growth factor-β (TGF-β) superfamily [6], is mainly synthesized by hepatic mesenchymal cells and released into the blood through autocrine or paracrine secretion [7]. Preclinical and clinical studies highlight that BMP9 influences the factors in MetS such as type 2 diabetes (T2DM) and NAFLD [8]. Luo et al. found that compared to healthy subjects, serum BMP9 levels were significantly lower in patients with T2DM [9]. Similarly, another study revealed that BMP9 levels decreased significantly in MetS patients than in healthy controls. Hao et al. found circulating BMP9 decreased in patients with T2DM and NAFLD [10]. Our previous study [11] showed BMP9 was significantly reduced in NAFLD mice, and supplement of BMP9 improved NAFLD by downregulating the expression of genes related to glucometabolism and lipometabolism, thereby reducing liver inflammation. All these findings hint BMP9 has the potential as a serological biomarker and new treatment for NAFLD and MetS. This trial intends to explore whether BMP9 can be used as a novel biomarker for severity of NAFLD and MetS using a population-based cohort study, to provide evidence for early diagnosis and intervention of high-risk individuals for NASH, thereby reducing the adverse outcomes of NAFLD.

## Methods

### Study population

A total of 263 adults who underwent FibroTouch testing in Shanghai East Hospital from February 2020 to December 2021 were included in this study after obtaining informed consent. This trial was approved by the Ethics Committee of Shanghai East Hospital ([2020] Research Approval No. 009). The inclusion criteria were as follows: (1) age: 18–75 years; (2) sufficient information such as history, clinical manifestations, physical signs and laboratory examination was available from the medical record database. The exclusion criteria were as follows: (1) alcohol consumption > 30 g/d for men and > 20 g/d for women; (2) the presence of conditions that may lead to hepatic steatosis, such as the use of special drugs, total parenteral nutrition, Inflammatory Bowel Disease (IBD), hypothyroidism, celiac disease and hypercortisolism; (3) other liver diseases, malignant tumors, active infection and severe mental or cognitive impairments; and (4) pregnant or lactating women.

MetS is a constellation of risk factors, and the main components of MetS were selected as follows in our study: (1) overweight and obesity: body mass index (BMI) > 25.0 kg/m^2^; (2) hyperglycemia: fasting plasma glucose (FPG) ≥ 6.1 mmol/L and/or 2 h plasma glucose (PG) ≥ 7.8 mmol/L or previously diagnosed T2DM; (3) dyslipidemia: triacylglycerol (TG) ≥ 1.7 mmol/L and high-density lipoprotein (HDL) ≤ 0.9 mmol/L (male), ≤ 1.0 mmol/L (female); and (4) hypertension: blood pressure ≥ 140/90 mmHg or use of medication for hypertension [12]. Since NAFLD has become the most common chronic liver disease in metabolic syndrome characterized by steatosis associated with inflammation, fibrosis and current researches demonstrated hyperuricemia promotes the development of steatosis [13, 14], it plays a more important role than blood pressure in NAFLD, and therefore, in our study, we used uric acid (UA) instead of blood pressure as one component of MetS.

All the participants were divided into NAFLD group and healthy controls according to the controlled attenuation parameter (CAP) of liver fat (CAP ≥ 240 dB/m indicated liver steatosis) by the FibroTouch detected diagnostic system and ultrasound [15, 16]. In NAFLD group, the patients were subsequently re-divided into NASH at-risk and NAFL group according to the results of blood liver function (alanine aminotransferase (ALT) > 40 U/L or aspartate aminotransferase (AST) > 35 U/L or γ-glutamyl transferase (GGT) > 45 U/L) [17].

### Anthropometric and hematological parameters

The following general details were collected: age, gender, height, weight, past medical, drinking and medication history and BMI was calculated using the equation: [BMI (kg/m^2^) = weight/height^2^]. Blood samples were obtained from the antecubital vein of each participant after 8 h of overnight fasting. Routine blood test was measured by LH-750 Blood Cell Analyzer (Beckman Coulter, Fullerton, CA, USA). Serum biochemistry, lipid profile, FPG and glycated hemoglobin (HbA1c) were measured by automated biochemical analyzer (AU5400; Olympus Corporation, Tokyo, Japan). According to the manufacturer’s instructions, serum BMP9 level was detected by enzyme-linked immunosorbent assay (ELISA) kit (Hengyuan Biotechnology co., Ltd, Shanghai, China) and calculated using a standard curve. Three separate plates (3 technical replicates) were run for each sample.

### Liver fat content and liver elasticity test

The liver stiffness measurement (LSM) values expressed in KPa, detected by FibroTouch (FIBROTOUCH-FT5000, HISKY MED, WX, China), were used to determine the degree of liver fibrosis, and the CAP values were expressed in dB/m to determine the degree of steatosis. FibroTouch was operated by an experienced ultrasound physician and applied to detect the liver stiffness and steatosis of all participants. An ultrasonic probe was placed in the area covering the seventh to the ninth intercostal spaces from the right anterior axillary line to the midaxillary line of the participants. Marks were left on uniform hepatic tissues of proper thickness that were free from either artery, bile ducts or cysts. The device was then switched to elastography mode, and participants were asked to hold their breath for 3 s. When the image became stabilized, LSM was conducted. The measurement was taken 10 times, and the median was considered the final value and was expressed as the value of elasticity (KPa). A reliable LSM was defined as more than 10 valid shots, a success rate of at least 60% and an interquartile range (IQR) < 33%. The fat attenuation parameter (FAP) value was also acquired. The results were defined as follows: LSM > 8.0 kPa implied the presence of abnormal liver elasticity; and CAP ≥ 240 dB/m implied hepatic steatosis: mild: 240 ≤ CAP < 265 dB/m; moderate: 265 ≤ CAP < 295 dB/m; and severe: CAP ≥ 295 dB/m.

### Statistical analysis

Continuous variables were described as mean ± standard deviation (SD) according to their data distribution. Independent samples *t*-test was selected for comparison between two groups. Multiple groups of independent samples were compared by one-way analysis of variance, and based on the results of one-way statistical analysis, a logistic regression analysis model was constructed to explore relevant risk factors, and *p* < 0.05 was considered to be statistically significant. All data analysis and graph drawing were performed by Statistical product and service solutions (SPSS) Statistics 20, R 3.6.3 (IBM, Armonk, NY) and GraphPad Prism 8 (GraphPad Software, San Diego, USA).

## Results

### Clinical characteristics and biochemical parameters in this study cohort

There were 166 subjects with an average age of 45.24 ± 14.32 years; among them, 62% (*n* = 103) were male, and 38% (*n* = 63) of them were female, with a sex ratio of 1.63:1. Based on the aforementioned grouping criteria, 66 (39.76%) were assigned to the healthy control group, 68 (40.96%) to the NAFL group and 32 (19.28%) to the NASH at-risk group. The comparison of clinical and biochemical parameters in each group was summarized elsewhere (Table 1). The characteristics of these three groups were different, except for ALB, PLT, TC. The NASH at-risk group had significantly higher levels of all other indicators, other than HDL and HbA1c. Significant differences were found in age (*p* < 0.01), height (*p* < 0.001), weight (*p* < 0.0001) and BMI (*p* < 0.0001) among the three groups.

**Table 1:** Clinical and biochemical characteristics of participants in healthy control, NAFL and NASH at-risk group (*n* = 166)

Group	Healthy control (*n* = 66)	NAFL (*n* = 68)	NASH at-risk (*n* = 32)	*F*	*p*
Age (year)	41.35 ± 13.75	46.73 ± 14.78	50.13 ± 13.78	4.88	0.009
Sex (male/female)	1.62/1	1.28/1	1.09/1	/	/
Height (cm)	164.74 ± 8.10	169.86 ± 8.09	170.09 ± 7.30	8.49	0.0003
Weight (kg)	57.21 ± 7.30	74.80 ± 13.12	76.16 ± 12.64	52.70	< 0.0001
BMI (kg/m^2^)	21.06 ± 1.99	25.82 ± 1.32	26.21 ± 3.35	56.12	< 0.0001
ALT (U/L)	16.30 ± 9.16	18.50 ± 7.05	44.69 ± 28.17	47.28	< 0.0001
AST (U/L)	18.95 ± 5.08	17.60 ± 3.99	34.03 ± 20.63	31.70	< 0.0001
GGT (U/L)	17.64 ± 11.74	23.49 ± 9.95	91.75 ± 83.62	46.27	< 0.0001
ALB (g/L)	47.40 ± 2.79	47.30 ± 2.65	46.63 ± 2.36	0.98	0.3762
PLT (10^9^/L)	235.61 ± 53.55	235.66 ± 46.95	229.00 ± 52.67	0.22	0.8023
TC (mmol/L)	4.72 ± 0.94	4.86 ± 0.98	5.15 ± 0.78	2.27	0.1070
TG (mmol/L)	1.23 ± 0.94	1.82 ± 1.55	2.33 ± 1.44	8.19	0.0004
HDL (mmol/L)	1.61 ± 0.54	1.26 ± 0.29	1.21 ± 0.23	16.61	< 0.0001
LDL (mmol/L)	2.88 ± 0.81	3.11 ± 0.78	3.30 ± 0.68	3.41	0.0355
FPG (mmol/L)	4.82 ± 0.49	5.57 ± 1.74	5.66 ± 1.43	7.01	0.0012
HbA1c (g/dL)	5.32 ± 0.30	5.85 ± 1.21	5.68 ± 0.43	7.07	0.0011
UA (μmol/L)	276.74 ± 66.85	351.12 ± 82.76	390.84 ± 58.47	32.01	< 0.0001
CAP (dB/m)	217.94 ± 14.11	275.49 ± 19.04	283.26 ± 22.07	225.29	< 0.0001
LSM (KPa)	5.50 ± 0.94	6.14 ± 1.43	7.70 ± 3.49	14.89	< 0.0001

*NAFL* nonalcoholic fatty liver, *NASH* nonalcoholic steatohepatitis, *BMI* body mass index, *ALT* alanine aminotransferase, *AST* aspartate aminotransferase, *GGT* γ-glutamyl transferase, *ALB* albumin, *PLT* platelets, *TC* total cholesterol, *TG* triacylglycerol, *HDL* high-density lipoprotein, *LDL* low-density lipoprotein, *FPG* fasting plasma glucose, *HbA1c* glycated hemoglobin, *UA* uric acid, *CAP* controlled attenuation parameter, *LSM* liver stiffness

### Low serum BMP9, BMI and male sex are associated with a higher risk of NAFLD

Logistic regression analyses revealed that BMP9, gender and BMI were significantly correlated with different stage of NAFLD (Table 2). In the NASH at-risk and NAFL groups, each 1-unit increase in BMP9 reduced the risk of NAFLD by 55% and 39%, respectively. Therefore, BMP9 could be considered a protective factor for the progression of NAFLD.

**Table 2:** Logistic regression analysis of risk factors for NAFLD

Variable	Dependent variable	Regression coefficient	OR	Stat	*p*
Intercept	2	19.74	/	6.72	0.0095
Intercept	1	4.12	/	0.43	0.5109
BMP9	2	-0.8	0.45 (0.34, 0.59)	33.72	< 0.0001
BMP9	1	-0.5	0.61 (0.48, 0.77)	17.73	< 0.0001
Sex	2	3.98	53.27 (5.29, 536.67)	11.38	0.0007
Sex	1	2.63	13.86 (2.21, 86.87)	7.88	0.005
BMI	2	1.28	3.59 (2.08, 6.19)	21	< 0.0001
BMI	1	1.23	3.43 (2.03, 5.79)	21.27	< 0.0001

2: NASH at-risk group *vs* healthy control, 1: NAFL group *vs* healthy control; *p* < 0.05 has statistical significance

Despite tremendous research advancements in NAFLD, our understanding of sex differences in NAFLD remains insufficient [18]. In our study, we found the risk of NAFLD was significantly higher in male than female in both the NASH at-risk group (OR 53.27; 95% CI 5.29–536.67) and the NAFL (OR, 13.86; 95% CI 2.21–86.87) group.

Overweight and obesity are associated with increased risk for NAFLD and MetS. As shown in Table 2, with each 1-unit increase in BMI, the risk increased 3.59-fold and 3.43-fold in the NASH at-risk (OR 3.59; 95% CI 2.08–6.19) and NAFL groups (OR 3.43; 95% CI 2.03–5.79), respectively.

### The relationship of BMP9 and different BMI, sex, age

**Fig. 1:**
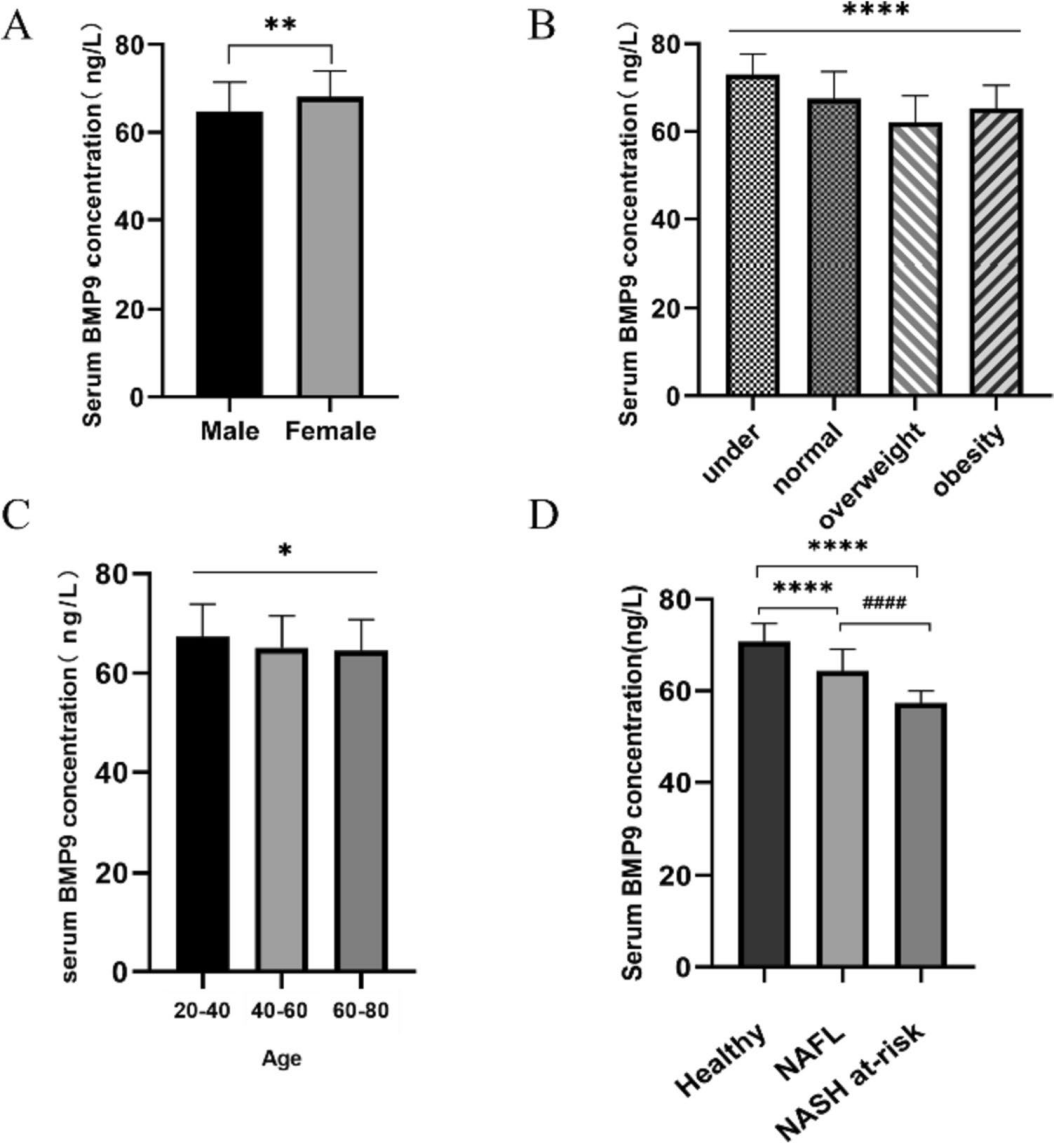
Serum BMP9 levels in different cohorts. **A** Serum BMP9 levels in male (*n* = 103) and female (*n* = 63). **B** Comparison of serum BMP9 levels among underweight group (*n* = 5), normal weight group (*n* = 105), overweight group (*n* = 46) and obese group (*n* = 10). **C** Serum BMP9 levels in various age groups: 20–40 years old (*n* = 75), 40–60 years old (*n* = 59), 60–80 years old (*n* = 32). **D** Comparison of BMP9 levels among healthy control (*n* = 66), NAFL group (*n* = 68) and NASH at-risk group (*n* = 32). *p < 0.05, **p < 0.01, ****p < 0.0001, ^####^*p* < 0.0001

BMI of all individuals was classified as underweight (BMI < 18.5 kg/m^2^), normal weight (18.5 ≤ BMI ≤ 24.9 kg/m^2^), overweight (25 ≤ BMI ≤ 29.9 kg/m^2^) and obesity (≥ 30 kg/m^2^) according to the World Health Organization 2000 criteria [19]. As shown in Fig. 1 and Table 3, serum BMP9 had a statistically significant difference between different BMI (*p* < 0.0001), sex (*p* < 0.01) and age (*p* < 0.05). Compared with male, females had higher BMP9 levels (Fig. 1A). In addition, BMP9 levels gradually decreased with increasing age (Fig. 1C).

**Table 3:** Association of serum BMP9 with age, sex and BMI

	Mean ± SD	Median (upper quartile, lower quartile)	Min, Max	*p*
Age				0.0133
20–40	67.40 ± 6.58	68.22 (62.60,72.20)	55.30,79.95	/
40–60	65.16 ± 6.38	65.37 (58.86,70.23)	52.39,76.88	/
60–80	64.66 ± 6.15	64.19 (59.84,68.45)	54.86,78.23	/
Sex				0.0016
Male	64.84 ± 6.57	64.11 (58.86,70.17)	52.39,79.95	/
Female	68.10 ± 5.90	68.87 (64.25,72.23)	55.17,78.76	/
BMI				< 0.0001
Under	73.00 ± 4.65	71.12 (69.08,77.87)	67.58,78.23	/
Normal	67.52 ± 6.14	68.16 (63.05,72.21)	54.70,79.95	/
Over	62.20 ± 5.90	60.91 (57.27,67.23)	52.39,75.44	/
Obesity	65.29 ± 5.23	65.42 (59.45,69.38)	58.65,73.02	/

*p* < 0.05 has statistical significance

### BMP9 was closely associated with the severity of NAFLD and could differentiate NASH at-risk from NAFLD

In order to clarify the relationship between blood BMP9 and NAFLD-related indexes, we conducted a correlation analysis between serum BMP9 levels and various NAFLD-related parameters. As shown in Table 4, the results indicated a statistically significant negative correlation between BMP9 concentration and the levels of ALT, AST, GGT (*p* < 0.0001), as well as with CAP (*p* < 0.0001) and LSM (*p* < 0.001). Although there was correlation direction between the levels of BMP9 and albumin (ALB) or platelets (PLT), no statistical differences were observed. Furthermore, the concentrations of BMP9 in healthy control group, NAFL group and NASH at-risk group were 70.32 ± 3.70 ng/L, 64.34 ± 4.76 ng/L and 58.13 ± 2.82 ng/L, respectively, as shown in Fig. 1D, and significant differences were observed among all the groups (all *p* < 0.0001). All these indicated that serum BMP9 was negatively associated with the severity of NAFLD and could differentiate NASH at-risk from NAFLD.

**Table 4:** Analysis of serum BMP9 and liver function-related indexes

Variable	1	2	3	4	5	7	8
1 BMP9							
Correlation							
Salience							
2 ALT							
Correlation	−0.362****						
Salience	< 0.0001						
3 AST							
Correlation	−0.302****	0.810					
Salience	< 0.0001	< 0.0001					
3 GGT							
Correlation	−0.379****	0.709	0.786				
Salience	< 0.0001	< 0.0001	< 0.0001				
4 ALB							
Correlation	0.042	−0.029	0.000	−0.048			
Salience	0.593	0.713	0.996	0.540			
5 PLT							
Correlation	0.068	0.062	−0.032	−0.074	0.119		
Salience	0.386	0.431	0.679	0.345	0.128		
6 CAP							
Correlation	−0.578****	0.383**	0.217**	0.319**	−0.123	−0.04	
Salience	< 0.0001	< 0.0001	0.005	< 0.0001	0.114	0.959	
7 LSM							
Correlation	−0.289***	0.385**	0.341**	0.365**	−0.66	−1.05	0.353
Salience	0.0002	< 0.0001	< 0.0001	< 0.0001	0.398	0.178	< 0.0001

*n* = 166; ****p < 0.0001

### BMP9 was closely associated with the severity of MetS components

**Fig. 2:**
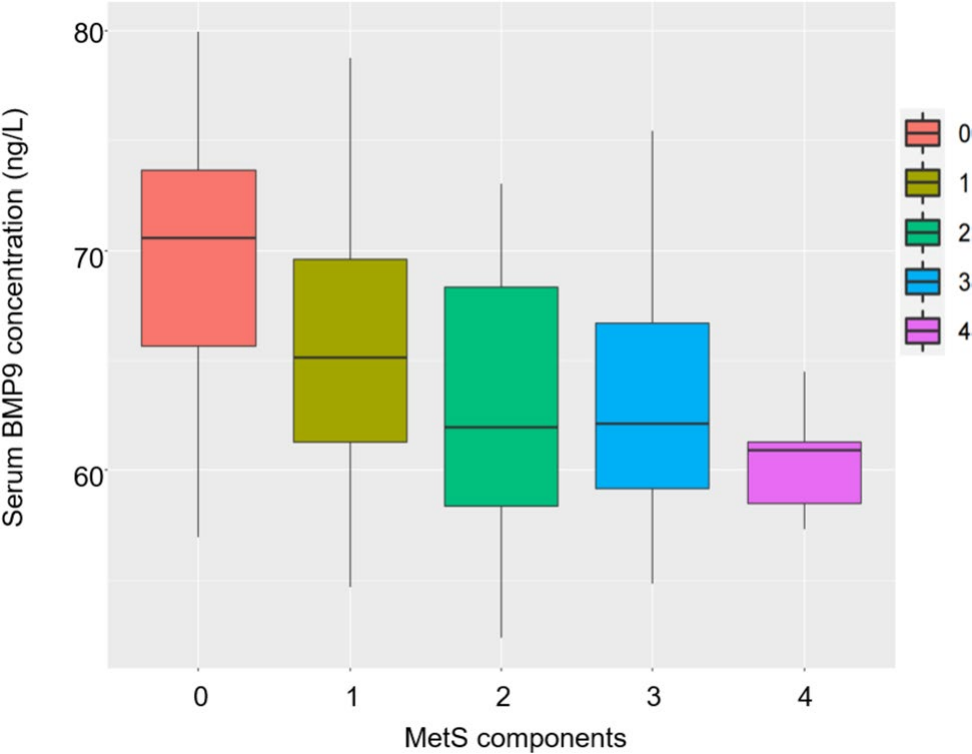
Relationship between serum BMP9 and the number of MetS components. 0 (*n* = 63), 1 (*n* = 41), 2 (*n* = 33), 3 (*n* = 24), 4 (*n* = 5)

It is widely accepted that there is a bidirectional relationship between NAFLD and various components of MetS. As mentioned above, BMP9 is associated with the severity and progression of NAFLD, and we further explored the relationship between BMP9 and parameters of MetS using correlation analysis. As revealed in Table 5, BMP9 had a negative correlation with concentrations of TG (*p* < 0.01), FPG, HbA1c (*p* < 0.05) and UA (*p* < 0.0001), while there was no significant correlation observed between BMP9 and total cholesterol (TC) or low-density lipoprotein (LDL). Additionally, it was observed that BMP9 was significantly correlated with HDL levels (*p* < 0.001). We further investigated the association between BMP9 and the number of MetS components. According to the number of MetS components, subjects were sorted and classified into groups 0 (*n* = 63), 1 (*n* = 41), 2 (*n* = 33), 3 (*n* = 24), 4 (*n* = 5). The results of stratified analysis showed that the mean (median) serum BMP9 concentration decreased with the increase in the number of MetS components (Fig. 2).

**Table 5:** Analysis of serum BMP9 and related indicators of glycolipid and FibroTouch detection

Variable	1	2	3	4	5	6	7
1 BMP9							
Correlation							
Salience							
2 TG							
Correlation	−0.229**						
Salience	0.003						
3 TC							
Correlation	−0.084	0.466					
Salience	0.282	< 0.0001					
4 LDL							
Correlation	−0.101	0.092	0.796				
Salience	0.195	0.239	< 0.0001				
5 HDL							
Correlation	0.294***	−0.371	−0.029	−0.157			
Salience	0.0001	< 0.0001	0.715	0.043			
6 FPG							
Correlation	−0.162*	0.256	0.173	0.120	−0.216		
Salience	0.037	0.001	0.026	0.124	0.005		
7 HbA1c							
Correlation	−0.153*	0.168	0.201	0.256	−0.172	0.802	
Salience	0.049	0.030	0.009	0.001	0.026	< 0.0001	
8 UA							
Correlation	−0.341****	0.296	0.143	0.155	−0.362	0.146	0.080
Salience	< 0.0001	< 0.0001	0.066	0.046	< 0.0001	0.061	0.304

*n* = 166; *p < 0.05; **p < 0.01; ***p < 0.001; ****p < 0.0001

## Discussion

NAFLD is a major chronic liver disease closely associated with obesity, insulin resistance (IR), T2DM, dyslipidemia and other MetS components, and it is the hepatic manifestation of MetS. The pathogenesis of NAFLD is complex whereby the historical two-hit hypothesis is now replaced by a multiple parallel hits hypothesis also considering the impact of an altered adipokine secretory pattern, inflammation, gut microbiota, nutritional factors, genetic and epigenetic factors, and IR on the development and progression of NAFLD [20]. NAFLD and MetS are closely related in both phenotype and pathogenesis. Coexistence of NAFLD and MetS should not be expected in every patient, one may precede the other, and common therapeutic modalities are beneficial for both entities.

BMP9, a hepatic cytokine that regulates blood glucose and lipid metabolism, is closely related to the pathogenesis of NAFLD and has a certain protective effect on NAFLD. It can inhibit hepatic lipid deposition, transform white adipose tissue (WAT) to brown adipose tissue and play a leptin-like role such as increasing energy expenditure to exert lipid-lowering effect [21]. Furthermore, BMP9 can play a hypoglycemic role in regulating glucose metabolism by increasing insulin sensitivity and stimulating the synthesis and secretion of insulin release [8]. Kuo et al. found that BMP9 induced WAT browning, inhibited high-fat diet-induced obesity and improved obesity-mediated IR and NAFLD in mice [22]. Our previous study also found the expression of BMP9 was decreased in NAFLD mice and exogenous supplementation of BMP9 improved the phenotype of NAFLD [11]. However, the relationship between serum BMP9 and the severity of MetS, NAFLD has not been explored thoroughly so far.

In the present study, we observed a negative correlation between the concentration of BMP9 and several indicators of glycolipid metabolism, including TG, FPG, HbA1c and BMI, while it was positively associated with HDL. This was consistent with other studies. Xu et al. found in middle-aged and older populations [23] as well as patients with T2DM and NAFLD [10], circulating BMP9 levels positively correlated with HDL and negatively correlated with markers of adiposity such as waist-to-hip ratio (WHR) or BMI, parameters of glucose metabolism (FPG and HbA1c) and TG. Animal studies also showed that upregulation of BMP9 levels inhibited the expression of lipogenic genes, ameliorated triglyceride accumulation in liver, decreased serum TC and TG [24], improved glucose tolerance, decreased FPG and alleviate IR in mice [11].

NAFLD is closely associated with obesity, and BMI is currently the most widely used anthropometric measure for assessing obesity worldwide and the most critical risk factor for NAFLD [25]. In our study, we discovered the risk of developing NAFLD increased along with elevated serum BMP9 and BMI, which is consistent with Younossi et al [26]. They also found that the prevalence of NAFLD was positively correlated with an increase in BMI. It is noteworthy that there was a significantly higher risk of NAFLD observed in men compared to women in our study. This has been confirmed in other studies that the prevalence of NAFLD is higher in men compared to women, and in women, the prevalence tends to increase after menopause, potentially due to the possible protective role of estrogen [27].

NAFLD has insidious progression and lacks specific symptoms. Noninvasive assessment of the severity of NAFLD is a general trend. Serum transaminase is often used as an index to detect NASH clinically, however, it does not reflect the course and prognosis of NAFLD accurately [28]. Another frequently used biomarker for NASH is cytokeratin 18 (CK18), which exhibits an overall sensitivity of 66% and specificity of 82% [29]. Nevertheless, inflammatory markers such as ferritin, high-sensitivity C-reactive protein (CRP), tumor necrosis factor and interleukins were demonstrated poor accuracy and specificity in recognizing NASH [30]. FibroTouch is now widely used in clinical practice as a noninvasive assessment method with excellent diagnostic performance for hepatic steatosis and fibrosis. The sensitivity of CAP values in diagnosing hepatic steatosis at stages S1, S2 and S3 was 81.5%, 86.7% and 100.0%, respectively, and the specificity was 80.6%, 91.7% and 96.5%, respectively [31]. Additionally, LSM outperformed other noninvasive fibrosis indices, namely fibrosis 4 (FIB-4), aspartate aminotransferase-to-platelet ratio index (APRI) and γ-glutamyl transferase-to-platelet ratio index (GPRI), demonstrating significant superiority, albeit second to liver biopsy [32]. Therefore, we employed FibroTouch in place of liver puncture to measure the degree of steatosis and fibrosis. In our research, we observed an upward trend in transaminases, CAP and LSM in patients with NAFLD compared to the healthy population. Moreover, we identified significant differences in BMP9 concentration among different liver enzyme levels, CAP and LSM. The current study also showed serum BMP9 levels differentiated NASH at-risk (58.13 ± 2.82 ng/L) from the other groups: healthy control (70.32 ± 3.70) and NAFL (64.34 ± 4.76 ng/L, *p* < 0.0001). Notably, BMP9 is widely involved in the function of various receptors associated with liver fibrosis promotion, downstream signaling molecules and the expression of target genes in hepatocytes [33], thus making it a valuable serum diagnostic indicator [34]. All these findings suggest that BMP9 has the potential to serve as a biomarker in identifying at-risk NASH patients.

Uric acid levels may play a role in MetS, and high uric acid level is independently associated with all components of MetS such as hypertension, obesity, high triglycerides, low HDL and elevated FPG [23]. In addition, another meta-analysis of prospective studies concluded that there is an independent linear relationship between increased UA levels and the occurrence of MetS [35, 36]. Therefore, in our study we used UA instead of hypertension as one of the MetS parameters, which are in line with previous studies [37]. Our results showed circulating BMP9 levels reduced with the number of MetS components. Xu et al. [23] also found that plasma BMP9 concentrations were significantly associated with MetS and circulating BMP9 levels reduced progressively with an increasing number of MetS components.

Although our study observed close association of serum BMP9 with the severity of MetS and NAFLD, there are some limitations including the small sample size, and the cross-sectional study design did not allow us to establish causality between serum BMP9 and NAFLD development. Future longitudinal studies with larger and more diverse participants are needed. Additionally, the absence of a liver biopsy-proven NAFLD cohort limits our comprehension for the connection of NASH and BMP9. Nonetheless, our findings suggest that BMP9 is highly has the potential to be used as a noninvasive biomarker for monitoring the severity and progression of NAFLD and MetS.

## Conclusion

The current study increases the limited evidence of the relationship between BMP9 and NAFLD as well as MetS, suggesting serum BMP9 was a new biomarker for noninvasive stratification of NAFLD and MetS. However, prospective longitudinal cohort studies with liver biopsy demonstrated are required with larger sample sizes.
